# Cerebro-Spinal Meningitis

**Published:** 1870-04

**Authors:** 


### *Cerebro-Spinal Meningitis.*

[Which of our correspondents will undertake to respond to this?—Ed.]

San Jacinto P.O., Houston Co., Minn., *Feb.* 20, 1870.

Messrs. Allen and Hay, M.D.—*Dear Sirs:* Permit me to suggest for your consideration the importance of enlightening the profession of medicine on the subject of that terrible disease, cerebro-spinal meningitis. I have seen five die with what I consider this complaint. Will you please to lay before the profession, *in extenso, all* that is known on the subject; causes, progress, lesions (post-mortem), diagnosis, prognosis and treatment. How distinguished from typhoid fever in the *beginning*. This is *most* important; then the steps taken, tell for or against all the way through. Respectfully, G. Jas. Sheldon, M.D.

